# Influence of plant diversity and elevated atmospheric carbon dioxide levels on belowground bacterial diversity

**DOI:** 10.1186/1471-2180-6-68

**Published:** 2006-07-27

**Authors:** Dominique Grüter, Bernhard Schmid, Helmut Brandl

**Affiliations:** 1 University of Zurich, Institute of Environmental Sciences, Winterthurerstrasse 190, CH-8057 Zurich, Switzerland

## Abstract

**Background:**

Changes in aboveground plant species diversity as well as variations of environmental conditions such as exposure of ecosystems to elevated concentrations of atmospheric carbon dioxide may lead to changes in metabolic activity, composition and diversity of belowground microbial communities, both bacterial and fungal.

**Results:**

We examined soil samples taken from a biodiversity × CO_2 _grassland experiment where replicate plots harboring 5, 12, or 31 different plant species had been exposed to ambient or elevated (600 ppm) levels of carbon dioxide for 5 years. Analysis of soil bacterial communities in these plots by temporal temperature gradient gel electrophoresis (TTGE) showed that dominant soil bacterial populations varied only very little between different experimental treatments. These populations seem to be ubiquitous. Likewise, screening of samples on a high-resolution level by terminal restriction fragment length polymorphism (T-RFLP) showed that increased levels of carbon dioxide had no significant influence on both soil bacterial community composition (appearance and frequency of operational taxonomic units, OTUs) and on bacterial richness (total number of different OTUs). In contrast, differences in plant diversity levels had a significant effect on bacterial composition but no influence on bacterial richness. Regarding species level, several bacterial species were found only in specific plots and were related to elevated carbon dioxide or varying plant diversity levels. For example, analysis of T-RFLP showed that the occurrence of *Salmonella typhimurium *was significantly increased in plots exposed to elevated CO_2 _(P < 0.05).

**Conclusion:**

Plant diversity levels are affecting bacterial composition (bacterial types and their frequency of occurrence). Elevated carbon dioxide does not lead to quantitative alteration (bacterial richness), whereas plant diversity is responsible for qualitative changes (bacterial diversity).

## Background

Knowledge on the relationship between plant communities and soil microbial communities is still lacking in large parts, although recent ecological research focuses on potentially beneficial effects of biodiversity on ecosystem processes, including the response of ecosystems to environmental changes such as increasing atmospheric carbon dioxide levels [1-3].

Soil microorganisms are the driving force behind soil organic matter transformations such as mineralization of organic compounds. These transformations are the basis of plant decomposition, soil aggregation, nutrient availability, soil fertility and, therefore in general, soil ecosystem functioning. However, these transformations might be significantly influenced by different levels of plant diversity which can affect soil microbial communities regarding e.g. population sizes, activities and taxonomic composition (see e.g. [4-8]. In addition, anthropogenic activities resulting in e.g. increasing atmospheric carbon dioxide concentrations might induce specific responses (stimulation, inhibition) of soil microbes (bacteria, fungi) possibly mediated via altered growth of the plant communities [9-12].

On both local and global scales, the wealth of soil microbial diversity is poorly appreciated and, therefore, the importance of soil organisms has been largely neglected [13]. A profound understanding of soil biodiversity and its relation to ecosystem functions is vital for long-term sustainable soils [14]. However, detailed knowledge on the control of ecosystem processes and functioning by this diversity is still lacking [15].

Soil microbial communities are characterized by two levels of diversity, namely genetic diversity and functional diversity: a high level of genetic diversity is found in many different types of soil (e.g. [16]). Recent detailed investigations based on molecular methods such as DNA-DNA-hybridization, 16S rRNA sequencing, PCR-based methods with primers derived from rRNA sequencing, fluorescence *in situ *hybridization (FISH), or immunological techniques revealed that soil microbial communities are composed of a vast variety of microorganisms resulting in complex microbial interactions and nutrient flows [17]. The composition of these communities is usually subject to seasonal fluctuations and may vary between different locations. In addition, organisms are not homogeneously distributed over the whole environment [18,19]. Regarding soil, it has been hypothesized that significant reductions in microbial diversity due to environmental changes are unlikely and that the genetic diversity does not represent a major factor that limits ecosystem functioning [15].

Elevated atmospheric CO_2 _can have indirect effects on soil microbial communities via altered plant inputs (litter, exudates, rhizodeposition). As result, soil microbial communities and their activities are stimulated: increased carbon flow might affect the portion of culturable soil bacteria and might favor fast growing organisms [20]. In turn, also the nitrogen flow (e.g. N-fixation) in soil ecosystems can be influenced by elevated CO_2 _[21], although nitrogen concentration in plant litter is not affected [22]. Therefore, microbial community composition and functional diversity are subject to changes under changing environmental conditions and the populations will adapt to the new conditions.

We examined soil samples from experimental grassland plots where plant communities of different species richness had been exposed for 5 years to ambient or elevated levels of carbon dioxide and assessed soil microbial community structure. The two main questions were: (i) what is the effect of elevated CO_2 _and plant diversity on bacterial richness? (ii) to which extent are treatment-induced effects reflected in changes in the structural composition of the soil bacterial community? An innovative feature of this study is the combination of an experimental-ecological approach and a microbiological approach to characterize the microbial populations involved. We determined the total number of soil microbial operational taxonomic units (OTUs) as a measure for "bacterial richness" as well as the dissimilitude of these OTUs as a measure for bacterial composition applying analyses of terminal restriction fragment length polymorphism (T-RFLP).

## Results

**Figure 1:**
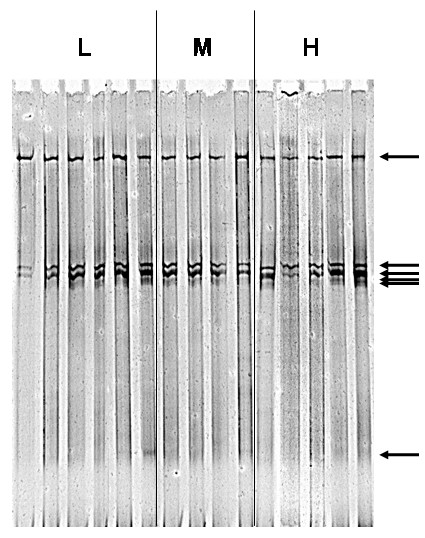
Example of TTGE band pattern (arrow: operational taxonomic unit, OTU) of DNA extracted from soil samples exposed to different levels of plant biodiversity and carbon dioxide. H: high diversity level (31 plant species); M: medium diversity level (12 plant species); L: low diversity level (5 plant species). Image was photographically enhanced using Photoshop.

Dominant soil microbial populations (as determined by TTGE) varied only very little between different soil samples (Fig. 1). Only a few bands were observed. The band patterns of the total community 16S rDNA showed that bacterial communities consisted of five to six bacterial groups that were present in all of the differently treated soils examined. These populations seemed to be ubiquitous and occurred in all samples, independently of experimental treatments or sampling location. Only very rarely additional OTUs were detected (e.g. lane 13). As a consequence, we found that soil bacterial community structure was only poorly resolved when analyzing bacterial populations by TTGE. In our case, the resolution of TTGE was too low for the monitoring of soil microbial diversity.

To improve analysis on a high resolution level, soil microbial populations were assessed by T-RFLP [23]. Different TRF'-types were applied, namely (i) 3'-BstU, (ii) 5'-BstU, (iii) 3'-MNL, and (iv) 5'-MNL (see Material & Methods). Combination (i), (ii) and (iii) yielded 20, 21 and 19 operational taxonomic units (OTUs), respectively, whereas 44 OTUs were obtained from combination (iv). Every of the four different TRF'-types applied may constitute on its own a possibility to determine soil microbial diversity. A combined analysis, however, allows more powerful statistical analysis and to monitor soil microbial diversity more comprehensively regarding the different OTUs present.

**Figure 2:**
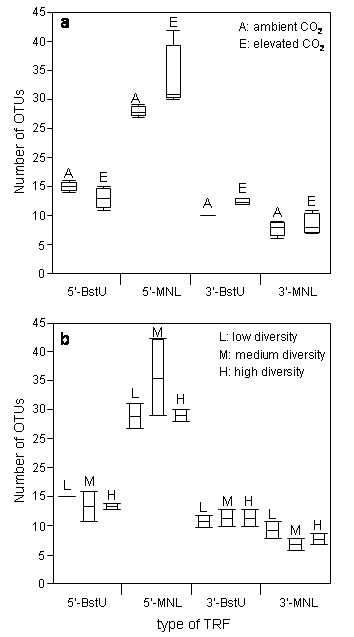
Number of operational taxonomic units (OTUs) observed in relation to different restriction enzyme/fluorescent label combinations. Boxes give median with upper line representing 75th percentile and lower line 25th percentile; whiskers extend to the most extreme data points with range not more than 1.5 times the interquartile range from the box. Number of OTU is a measure for "bacterial richness". a) elevated carbon dioxide in comparison to ambient levels; b) three different plant diversity levels.

However, combining the information obtained from all assays (enzyme/label combinations) showed that elevated carbon dioxide levels does not significantly influence the number of soil microbial community OTUs (= "bacterial richness") (Fig 2a). Moreover, also different plant diversity levels showed no significant effect on this bacterial richness measure (Fig. 2b). No interaction was detected between carbon-dioxide and diversity treatments.

**Figure 3:**
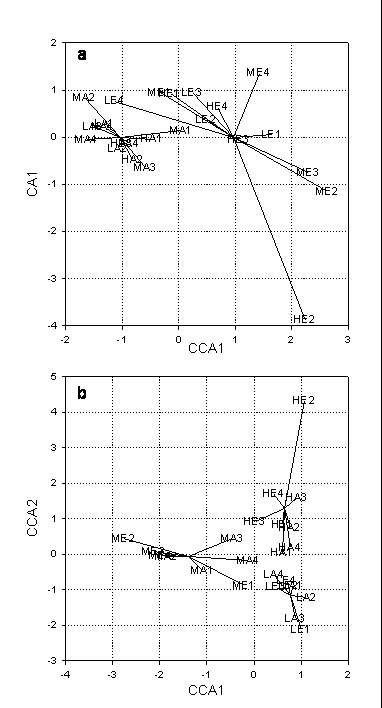
Canonical correspondence analysis of plots exposed to different carbon dioxide levels (a); plots exposed to different plant diversity levels (b). A: ambient carbon dioxide; E: elevated carbon dioxide; H: high plant diversity; M: medium plant diversity; L: low plant diversity; numbers 1, 2, 3, and 4: four replicates.

To analyze bacterial composition, all band patterns of the different enzyme/label combinations were analyzed in combination by canonical correspondence analysis. Occurrence of OTUs within the four replicates of each treatment combination was analyzed separately, but additionally also the frequency of occurrence of OTUs within the four replicates was assessed. As shown in Fig. 3b, plant diversity had a significant (P < 0.05) effect on bacterial composition. Three distinct separate clusters can be observed, each related to one of the three different plant diversity levels. In contrast, elevated carbon dioxide did not affect bacterial composition (Fig. 3a). Although a clustered pattern was observed, a significant difference was not detectable.

All TRFs were compared with the TAP-database. Only bacterial strains simultaneously matching maximally two types of enzyme/label combinations were considered (Table 1). No strains simultaneously matching three or four enzyme/label combinations were detected. In several cases, results yielded more than one bacterial species for the same enzyme/label combination. The frequency of occurrence of the different combinations of TRFs and bacteria belonging to them was variable. Several bacterial strains were found only in specific plots and were obviously related to elevated carbon dioxide or varying plant diversity levels: *Escherichia coli *and *Ferrobacterium limneticum *were detected only in three samples, all showing a high plant diversity level. The patterns referring to the unidentified strain from Lake Gossenkoellesee was found only in samples with medium plant diversity (12 different plant species). *Clostridium perfringens*, *Sulfobacillus disulfidooxidans*, *Kitasatospora paracochleata*, *Kitasatospora melanogena *and *Kitasatospora kifuense *were found only in plots with elevated carbon dioxide levels. Furthermore, analyses of the different TRF patterns by logistic regression showed that the probability of occurrence of *Salmonella typhimurium *in plots treated by elevated CO_2 _was significantly increased (P < 0.05).

## Discussion

Several studies found a positive relationship between elevated CO_2 _and bacterial richness [11], whereas others found a negative effect [24]. Therefore, results show a certain inconsistency [25,26]. In our case, considering the negligible effect of elevated carbon dioxide and of different plant diversity levels on the number of OTUs detected, it can be concluded that both experimental treatments had no effect on bacterial richness. Furthermore, elevated carbon dioxide concentrations did not affect soil microbial composition as also reported by Griffiths *et al*. [27] or Zak et al [28]. In contrast, aboveground plant diversity significantly affected belowground bacterial composition. These findings suggest that the soil microbial composition is mainly related to plant diversity (assuming that different plant species might harbor specific rhizospheric microbial populations) rather than altered soil carbon fluxes induced by elevated atmospheric CO_2 _and subsequently increased photosynthetic activities.

Our analyses showed that increased levels of carbon dioxide had no influence on soil microbial community composition (Fig. 3a). Canonical correspondence analysis (CCA) showed no statistical difference between plots at ambient CO_2 _level compared with plots with elevated CO_2 _level. Differences in rhizosphere carbon allocation (and subsequent alterations of soil microbial communities) have been postulated and observed when plants were exposed to increased CO_2 _levels in other studies, e.g. changed quality of litter, increase in root exudates and stimulation of rhizodeposition [29,30]. Consequently, effects on soil microbial community composition might occur [9]. However, in our case the aboveground exposure of plants to elevated carbon dioxide was not reflected in a belowground change of bacterial composition.

In contrast to the CO_2 _treatment, plant diversity had a significant effect on belowground microbial community composition (Fig. 3b). That is, on a genetic level, bacterial community structure in soil can be differentiated in relation to different plant diversity levels. This is in agreement with studies reported earlier on the functional microbial diversity in soil samples exposed to different plant diversity levels [4]. The differentiation between the three different plant diversity levels on the basis of the soil bacterial composition suggests that all three plant diversities exhibit their own bacterial environment.

The land where our experimental plots have been set up, has a long-time record as pasture for cows. Therefore, the occurrence of *Escherichia coli *and *Salmonella *sp. is not surprising since these organisms easily originate from cowpats as shown by Muirhead *et al*., [31] and Johannessen *et al*. [32]. Anoxic micro-habitats might occur in soil allowing the existence and survival of anaerobes such as Clostridia. In addition, *Clostridium *is an spore-forming organism able to form spores which can easily survive in soil for prolonged time periods. Since *Ralstonia solanacearum *is commonly found in soil as plant pathogen but also free-living when host plants are absent [33], the occurrence is not unusual. Therefore, we conclude that our findings reflect the true state of the soil investigated.

As stated by other authors [34], soil type might be the key determinant for soil microbial communities. Since the soil type was the same throughout all experimental plots, we believe that the differences we have observed reflect the true state of the soil depending on the different treatments e.g. elevated CO_2 _or plant diversity.

## Conclusion

In summary, plant diversity levels are affecting bacterial composition (bacterial types and their frequency of occurrence). Elevated carbon dioxide does not lead to quantitative alteration (bacterial richness), whereas plant diversity is responsible for qualitative changes (bacterial diversity).

## Methods

### Site description

Soil samples were collected from a nutrient-poor, calcareous experimental grassland in northwestern Switzerland (for a more detailed site description see [35][36][37]). The field site is located on a southwest-facing slope in the Jura mountains of Switzerland near the village of Nenzlingen (47°33'N 7°34'E). Detailed information on soil characteristics have been already published elsewhere. As described by Niklaus *et al*. [38], "the Rendzina-type soil, which is typical for these habitats, consists of a 10 to 15 cm neutral to slightly basic (pH approx. 7.8) silty clay loam top soil and is underlain with calcareous debris. In the top 10 cm, the horizon, where most of the fine roots occur, organic C and N are approx. 3.9% and 0.33%, respectively". Typically, the climate in this area is characterized by cold wet springs, warm drought-prone summers, pleasant falls, and moderate winters [39].

### Experimental design

Twenty-four plots (1.27 m^2 ^each) received factorial combinations of two carbon dioxide treatments at three plant species diversity levels. CO_2 _treatments were (i) 12 open-top, open-bottom chambers with ambient CO_2 _(approx. 350 ppm) ["A"]; 12 open-top, open-bottom chambers with elevated CO_2 _(approx. 600–650 ppm) ["E"] using the Screen-Aided CO_2 _Control system (SACC) for carbon dioxide exposure [35]. The plant species diversity levels were (i) 31 species ("high" diversity level) ["H"], 12 species ("medium" diversity level, all species also present in H) ["M"], and 5 species ("low" diversity level, all species also present in M and H) per plot ["L"]. Plants were selected from three functional groups grasses, herbs and legumes [36]. Diversity treatments were established in September 1993, prior to the onset of CO_2 _treatment which started in early April 1994. Each treatment combination (carbon dioxide level × plant diversity level) was replicated four times. The treatment combinations are accordingly named "HA", "MA", "LA", "HE", "ME", and "LE".

### Soil sampling

Soil sampling took place in spring 1999. Six samples were taken from the top layer (0–10 cm) from each plot. Soil coring was done with a core bit which was sterilized by flaming with ethanol prior to every sampling. Three samples were pooled in a sterile plastic tube (Greiner AG, Kremsmünster, Austria), kept on ice and immediately frozen at -80°C after returning to the laboratory, resulting in two independent samples from each plot.

### DNA extraction

Prior to DNA extraction, all samples were lyophilized over night and ground in sterile plastic tubes with sterile glass beads. Total community DNA was extracted using the Ultra Clean Soil DNA Kit (MO BIO Laboratories, Inc.), following the manufacturer's instructions. DNA extracts were stored at -20°C.

### Temporal temperature gradient gel electrophoresis (TTGE)

Samples were prepared by amplifying approximately a 500-bp piece of bacterial 16S rRNA gene, using the bacterial universal primers 341F (5'CCTACGGGAGGCAGC AG-3') and 907R-GCclamp (5'CGCCCGCCGCGCGCGGCGGGCGGGGCGGG GGCACGGGGGGCCGTCAAATTCMTTTRAGTTT-3'). TTGE was performed in 1.25 × TAE buffer (1 × TAE corresponds to 40 mM Tris-acetate, 20 mM acetic acid, 1 mM EDTA, pH 8.3) for 7 h. For analysis of bacterial 16S rDNA fragments, gels were run at 60 V over a temperature range of 60–70°C increasing at a rate of 1.4°C/h.

### Terminal restriction fragment length polymorphism (T-RFLP)

16S rRNA genes were amplified using the eubacterial universal primer combination of 6-carboxyfluorescein (FAM)-labeled primer 27F (5'-AGAGTT-TGA-TCC-TGG-CTC-AG-3') and 6-carboxyfluorescein (JOE)-labeled primer 1525R (5'-AAG-GAG-GTG-WTC-CAR-CC-3'). PCR amplification parameters were as follows: 94°C and 2 min of initial melt and polymerase activation; 35 amplification cycles of 94°C, 30 sec; 55°C, 30 sec; and 72°C, 2.5 min; and a final extension at 72°C for 5 min in a thermocycler (Biometra). The standard reaction mixture contained in a total volume of 100 μl, 1 × JumpStart ReadyMix Taq (Sigma), 0.4 μM concentration of each primer (Microsynth GmbH, Balgach, Switzerland) and genomic DNA (< 60 ng). The fluorescently labeled products were purified with the MinElute Gel Extraction Kit (Qiagen, Hilden, Germany) according to the protocol provided by the supplier and were digested for 6 h at 37°C with restriction enzyme MNL1 (MBI Fermentas, St. Leon-Rot, Germany) or, alternatively, for 6 h at 60°C with the restriction enzyme BstU1 (New England Biolabs, Beverly, USA). T-RFLP analysis was carried out on an ABI 310 genetic analyzer (Perkin-Elmer, Foster City, Calif.) with Genescan software and an internal size standard (ROX 500). Cutoffs were applied (5 base pairs to 480 base pairs). Peaks with < 5% of maximum intensity were neglected. Alignment was done using standards size Gene scan 500. Position tolerance was < 0.5%. All together, FAM-labeled 5'-ends and JOE-labeled 3'-ends of the PCR-products cut by two different restriction enzymes resulted in four different types of terminal restriction fragments (TRF), namely (i) 3'-BstU, (ii) 5'-BstU, (iii) 3'-MNL, and (iv) 5'-MNL. Resulting TRFs were compared with the results of a virtual search for TRFs with the T-RFLP analysis program (denoted as TAP T-RFLP) [40].

### Statistical analysis

Modeling and canonical correspondence analysis were done with the open source software package R [41]. In particular, canonical correspondence analysis (CCA) – a multivariate analysis method derived from correspondence analysis – was performed to compare the similarity of band patterns obtained from T-RFLP. All data were checked for normal distribution and transformed if necessary.

## Authors' contributions

All experimental work was carried out by DG under the supervision of BS and HB. BS provided also statistical help. HB was the principal investigator. DG and HB wrote the manuscript. All authors read and approved the final manuscript.

## Figures and Tables

**Table 1:** Organisms identified by double matches of restriction enzyme/fluorescent label combinations

Enzyme/label combination	Fragment	Organism identified, species	strain; type culture collection number	number of plots (out of 24) where strain was detected
Bst-FAM × BstU-JOE	226/173	*Clostridium botulinum*	202F; ATCC 23387	5
		*Clostridium butyricum*	E.VI.3.6.1.; NCIMB 8082	
		*Clostridium butyricum*	MMP3; DSM 2478	
		*Clostridium butyricum*	ATCC 43755	
	236/173	*Bacillus subtilis*		7
		unidentified species	16SX-1	
		unidentified species	16SX-2	
BstU-JOE × MNL-JOE	171/93	*Ralstonia solanacearum*	ACH 0732	24
	171/93	*Piscirickettsia salmonis*		5
	173/93	*Pirellula *sp. (uncultered)	clone 5H12	5
		*Thermoanaerobacterium saccharolyticum*	B6A-RI	
	275/93	*Ferribacterium limneticum*	cda-1	3
		*Escherichia coli *chloroplast of *Marchantia polymorpha*		
BstU-JOE × MNL-FAM	173/210	symbiont of *Crithidia *sp.		24
	275/205	unidentified strain from Gossenköllesee		3
	275/211	*Azoarcus *sp.	PH002	7
		*Azoarcus *sp.	CR23	
		*Azoarcus *sp.	FL05	
		unidentified species	clone A1–13	
		unidentified species	cloneSJA-62	
	275/217	*Salmonella typhimurium*	E10 NCTC 8391	15
	275/226	unidentified species	clone SJA-47	2
MNL-JOE × BstU-FAM	226/93	*Clostridium algidicarnis*	NCFB 2931 (T)	1
BstU-FAM × MNL-FAM	103/211	unidentified species	clone SJA-112	11
	219/426	*Frankia *sp.	L27	1
		*Kibdelosporangium aridum *subsp. *aridum*		
		*Mycobacterium asiaticum*	N6IH; ATCC 25276	
	221/129	*Nocardia crassostreae*	RB1	22
		*Nocardia crassostreae*	OB3	
	221/183	*Sulfobacillus disulfidooxidans*	SD-11	12
		*Kitasatospora paracochleata*	14769	
		*Kitasatospora melanogena*	JCM 3337	
		*Kitasatospora kifuense*	JCM 9081	
	236/138	*Leuconostoc citreum*	NCDO 2787	7
		*Leuconostoc lactis*	ATCC 19256	
		*Leuconostoc mesenteroides *subsp. *mesenteroides*	ATCC 8293	
	388/210	*Oxalobacter formigenes*	BLISS	12
	390/211	*Pseudomonas *sp.	B13	11
MNL-FAM × MNL-JOE	134/93	*Clostridium perfringens*	CPN50	2

## References

[B1] Schmid B, Joshi J, Schläpfer F, Kinzig A, Pacala S, Tilman D (2002). Empirical evidence for biodiversity-ecosystem functioning relationships. The Functional Consequences of Biodiversity: Empirical Progress and Theoretical Extensions.

[B2] Kohut R (2003). The long-term effects of carbon dioxide on natural systems issues and research needs. Environ Int.

[B3] Zavaleta ES, Shaw MR, Chiariello NR, Mooney HA, Field CB (2003). Additive effects of simulated climate changes, elevated CO_2_, and nitrogen deposition on grassland diversity. Proc Natl Acad Sci USA.

[B4] Stephan A, Meyer AH, Schmid B (2000). Plant diversity positively affects soil bacterial diversity in experimental grassland ecosystems. J Ecol.

[B5] Kowalchuk GA, Buma DS, de Boer W, Klinkhamer PGL, van Veen JA (2002). Effects of above-ground plant species composition and diversity on the diversity of soil-borne microorganisms. Antonie van Leeuwenhoek.

[B6] de Deyn GB, Raaijmakers CE, Van der Putten WH (2004). Plant community development is affected by nutrients and soil biota. J Ecol.

[B7] Bartelt Ryser J, Joshi J, Schmid B, Brandl H, Balser T (2005). Carryover effects of soil inocula from a plant biodiversity experiment on subsequent plant growth. Perspect Plant Ecol Evol Syst.

[B8] Bonkowski M, Roy J (2005). Soil microbial diversity and soil functioning affect competition among grasses in experimental microcosms. Oecologia.

[B9] Sadowski MJ, Schortemeyer M (1997). Soil microbial responses to increased concentrations of atmospheric CO_2_. Global Change Biol.

[B10] Jones TH, Thompson LJ, Lawton JH, Bezemer TM, Bardgett RD, Blackburn TM, Bruce KD, Cannon PF, Hall GS, Hartley SE, Howson G, Jones CG, Kampichler C, Kandeler E, Ritchie DA (1998). Impacts of rising atmospheric carbon dioxide on model terrestrial ecosystems. Science.

[B11] Marilley L, Hartwig UA, Aragno M (1999). Influence of an elevated atmospheric CO_2 _content on soil and rhizosphere bacterial communities beneath *Lolium perenne *and *Trifolium repens *under field conditions. Microb Ecol.

[B12] Montealegre CM, van Kessel C, Blumenthal JM, Hur HG, Hartwig UA, Sadowsky MJ (2000). Elevated atmospheric CO_2 _alters microbial population structure in a pasture ecosystem. Global Change Biol.

[B13] Wall DH, Lynch JM, Balazs E, Galante E, Lynch JM, Schepers JS, Toutant JP, Werner D, Werry PATJ (2000). Soil biodiversity and ecosystem functioning. Biological Resource Management Connecting Science and Policy.

[B14] Mooney HA, Cushman JH, Medina E, Sala OE, Schulze ED, Mooney HA, Cushman JH, Medina E, Sala OE, Schulze ED (1996). What we have learned about the ecosystem functioning of biodiversity. Functional Roles of Biodiversity – A Global Perspective.

[B15] Balser TC, Kinzig AP, Firestone MK, Kinzig A, Pacala S, Tilman D (2001). Linking soil microbial communities and ecosystem functioning. The Functional Consequences of Biodiversity: Empirical Progress and Theoretical Extensions.

[B16] Torsvik V, Goksoyr J, Daae FL (1990). High diversity in DNA of soil bacteria. Appl Environ Microbiol.

[B17] Torsvik V, Ovreas L (2002). Microbial diversity and function in soil: from genes to ecosystems. Curr Opin Microbiol.

[B18] Nunan N, Wu KJ, Young IM, Crawford JW, Ritz K (2003). 2003. Spatial distribution of bacterial communities and their relationships with the micro-architecture of soil. FEMS Microbiol Ecol.

[B19] Ranjard L, Richaume A (2001). Quantitative and qualitative microscale distribution of bacteria in soil. Res Microbiol.

[B20] Paterson E, Hall JM, Rattray EAS, Griffiths BS, Ritz K, Killham K (1997). Effect of elevated CO_2 _on rhizosphere carbon flow and soil microbial processes. Global Change Biol.

[B21] Soussana JF, Hartwig UA (1996). The effects of elevated CO_2 _on symbiontic N_2 _fixation: a link between the carbon and nitrogen cycles in grassland ecosystems. Plant Soil.

[B22] Hartwig UA, Lüscher A, Daepp M, Blum H, Soussana JF, Nösberger J (2000). Due to symbiotic N_2 _fixation, five years of elevated atmospheric pCO_2 _had no effect on the N concentration of plant litter in fertile, mixed grassland. Plant Soil.

[B23] Liu WT, Marsh T, Cheng H, Forney LJ (1997). Characterization of microbial diversity by determining terminal restriction fragment length polymorphisms of genes encoding 16S rRNA. Appl Environ Microbiol.

[B24] Hodge A, Paterson E, Grayston SJ, Campbell CD, Ord BG, Killham K (1998). Characterisation and microbial utilisation of exudate material from the rhizosphere of *Lolium perenne *grown under CO_2 _enrichment. Soil Biol Biochem.

[B25] Whipps JM (1985). Effect of CO_2 _concentration on growth, carbon distribution and loss of carbon from the roots of maize. J Exp Bot.

[B26] O'Neill EG, Luxmoore RJ, Norby RJ (1987). Elevated atmospheric CO_2 _effects on seedling growth, nutrient uptake, and rhizosphere bacterial populations of *Liriodendron tulipifera *L. Plant Soil.

[B27] Zak DR, Ringelberg DB, Pregitzer KS, Randlett DL, White DC, Curtis PS (1996). Soil microbial communities beneath *Populus grandidentata *crown under elevated atmospheric CO_2_. Ecol Appl.

[B28] Fangmeier A, De Temmerman L, Mortensen L, Kemp K, Burke J, Mitchell R, van Oijen M, Weigel HJ (1999). Effects on nutrient and on grain quality in spring wheat crops grown under elevated CO_2 _concentrations and stress conditions in the European, multiple-site experiment 'ESPACE-wheat'. Eur J Agron.

[B29] Griffiths BS, Ritz K, Ebblewhite N, Paterson E, Killham K (1998). Ryegrass rhizosphere microbial community structure under elevated carbon dioxide concentrations, with observations on wheat rhizosphere. Soil Biol Biochem.

[B30] Pendall E, Mosier AR, Morgan JA (2004). Rhizodeposition stimulated by elevated CO2 in a semiarid grassland. New Phytol.

[B31] Muirhead RW, Collins RP, Bremer PJ (2006). Numbers and transported state of Escherichia coliin runoff direct from fresh cowpat under simulated rainfall. Lett Appl Microbiol.

[B32] Johannessen GS, Froseth RB, Solemdal L, Jarp J, Wasteson Y, Rorvik LM (2004). Influence of bovine manure as fertilizer on the biological quality of organic Iceberg lettuce. J Appl Microbiol.

[B33] Genin S, Boucher C (2004). Lessons learned from the genome analysis of Ralstonia solanacearum. Ann Rev Phytopathol.

[B34] Bossio DA, Girvan MS, Verchot L, Bullimore J, Borelli T, Albrecht A, Scow KM, Ball AS, Pretty JN, Osborn AM (2000). Soil microbial community response to land use change in an agricultural landscape of Western Kenya. Microb Ecol.

[B35] Leadley PW, Niklaus P, Stocker R, Körner C (1997). Screen-aided CO_2 _control (SACC): a middle ground between FACE and open-top chambers. Acta Oecol.

[B36] Leadley PW, Niklaus P, Stocker R, Körner C, Körner C, Bazzaz FA (1996). Effects of elevated CO_2 _on plant species dominance in a highly diverse calcareous grassland. Carbon Dioxide Populations and Communities.

[B37] Niklaus PA, Leadley PW, Stöcklin J, Körner C (1998). Nutrient relations in calcareous grassland under elevated CO_2_. Oecologia.

[B38] Niklaus PA, Alphei J, Ebersberger D, Kampichler C, Kandeler A, Tscherko D (2003). Six years of ins situ CO_2 _enrichment evoke changes in soil structure and soil biota of nutrient-poor grass land. Global Change Biology.

[B39] Leadley PW, Niklaus PA, Stocker R, Körner C (1999). A field study of the effects of elevated CO_2 _on plant biomass and community structure in a calcareous grassland. Oecologia.

[B40] Marsh TL, Saxman P, Cole J, Tiedje J (2000). Terminal restriction fragment length polymorphism analysis program, a web-based research tool for microbial community analysis. Appl Environ Microbiol.

[B41] R Development Core Team 2004 R: A language and environment for statistical computing. Vienna Austria: R Foundation for Statistical Computing.

